# Efficacy and Safety of Two Chondroprotective Supplements in Patients With Knee Osteoarthritis: A Randomized, Single-Blind, Pilot Study

**DOI:** 10.7759/cureus.57579

**Published:** 2024-04-04

**Authors:** Piercarlo Minoretti, Andrés Santiago Sáez, Miryam Liaño Riera, Manuel Gómez Serrano, Ángel García Martín

**Affiliations:** 1 Occupational Health, Studio Minoretti, Oggiono, ITA; 2 Legal Medicine, Hospital Clinico San Carlos, Madrid, ESP; 3 Legal Medicine, Psychiatry, and Pathology, Complutense University of Madrid, Madrid, ESP; 4 Legal Medicine, Psychiatry and Pathology, Complutense University of Madrid, Madrid, ESP

**Keywords:** nutraceuticals, chondroprotection, adropin, pain, knee, osteoarthritis, phytomedicine

## Abstract

*Background*: Hyaluronic acid (HA), glucosamine (Glc), and chondroitin sulfate (CS) are key ingredients commonly incorporated into dietary chondroprotective supplements for the management of osteoarthritis (OA). Despite their widespread use, there is a paucity of published data regarding their efficacy and safety, necessitating rigorous investigation in clinical settings. To address this knowledge gap, we conducted a randomized, single-blind pilot study to evaluate the effects of two commercially available multi-ingredient supplements on patients with mild-to-moderate knee OA.

*Methods: *A total of 51 patients diagnosed with mild-to-moderate knee OA were enrolled in a four-week randomized study and allocated equally (1:1:1 ratio) into three groups: a control group (n = 17) that received no treatment, an HA group (n = 17) given Syalox^®^ 300 Plus (1 tablet/day) containing HA (300 mg) and *Boswellia serrata* extract (100 mg), and a Glc + CS group (n = 17) given Cartijoint^®^ Forte (1 tablet/day) containing Glc (415 mg), CS (400 mg), and curcuminoids from rhizomes of *Curcuma longa* L (50 mg).Physicians conducting evaluations were blinded to group assignments, whereas patients were not. All participants underwent assessments of pain relief, functional capacity improvement, and serum adropin levels, an emerging biomarker of knee OA, at baseline and after the four-week intervention period.

*Results*: Both the HA and the Glc + CS groups exhibited improvements at the end of the study relative to baseline, with statistically significant differences (p < 0.05) observed in pain at rest, pain during movement, range of motion, and the overall Western Ontario and McMaster Universities Osteoarthritis Index (WOMAC) scores, including its pain, stiffness, and physical function subscales. Notably, the HA group outperformed the Glc + CS group in the alleviation of pain at rest, pain during movement, and on the WOMAC pain subscale, with all differences being statistically significant (p < 0.05). Additionally, both groups showed a significant elevation in serum adropin levels from baseline (p < 0.05), with the HA group experiencing a more substantial increase when compared to the Glc + CS group (p < 0.05). Both supplements showed a limited number of treatment-emergent adverse events.

*Conclusion*: Oral supplementation with either HA or Glc + CS demonstrated potential benefits for managing symptoms of mild-to-moderate knee OA. Notably, HA supplementation was associated with greater improvements in pain relief and higher elevations in serum adropin levels compared to Glc + CS supplementation. However, larger-scale and longer-term studies are necessary to further evaluate the safety and efficacy of these dietary supplements within the clinical management arsenal for knee OA.

## Introduction

Knee osteoarthritis (OA) is a prevalent degenerative joint disorder characterized by the gradual deterioration of articular cartilage integrity [1]. According to data from 2020, the global prevalence of knee OA is estimated to be 16.0% among individuals aged 15 years and older and 22.9% among those aged 40 years and older. Consequently, approximately 654.1 million individuals worldwide aged 40 years and older are affected by knee OA [2]. The pathogenesis of knee OA is multifactorial, involving a dynamic interaction among mechanical trauma, repetitive joint stress, and degenerative changes associated with aging or comorbid conditions [3]. These factors synergistically induce structural and functional alterations within the joint, culminating in the hallmark symptoms of pain, stiffness, and reduced ability to perform daily activities [3]. The traditional approach to managing OA primarily aims at alleviating symptoms, with an emphasis on reducing pain [4]. Clinical guidelines advocate for the administration of oral non-steroidal anti-inflammatory drugs (NSAIDs) for individuals experiencing continuous discomfort [5]. Despite NSAIDs being the cornerstone of pharmacological treatment for OA, their use is linked to a range of side effects, including gastrointestinal bleeding, cardiovascular complications, and possible kidney damage [6,7]. As a result, there is an increasing interest in investigating the potential of chondroprotective nutraceuticals as complementary or alternative options to conventional drug therapy [8,9].

Hyaluronic acid (HA) [10], glucosamine (Glc) [11], and chondroitin sulfate (CS) [12] are key ingredients frequently included in nutraceutical formulations aimed at promoting joint health. These substances are recognized for their chondroprotective qualities, suggesting their ability to support cartilage maintenance and repair [10]. A prior double-blind, placebo-controlled study evaluated the effects of an oral supplement containing low molecular weight HA, Glc, and CS on individuals with knee OA [13]. The supplementation resulted in significant pain relief, reduction of stiffness, enhancement of physical function, and overall quality of life [13]. Furthermore, an observational study examined the effectiveness of a dietary supplement that combined hydrolyzed collagen, CS, and Glc, among other ingredients, in reducing pain caused by OA [14]. Participants experienced notable pain relief and functional enhancements after 3 and 6 months of the intervention, respectively, without significant adverse effects [14].

Despite the promising outcomes observed in several studies, the literature is not consistent regarding the efficacy of nutraceuticals in OA. While some research indicated a degree of pain alleviation and functional enhancement surpassing that of placebos, other studies failed to demonstrate any substantial advantage [15,16]. Moreover, there is a lack of consensus on the ideal dosage, formulation, and long-term implications of these supplements' usage [16]. Given the complex pathogenesis of osteoarthritis, the combined use of various nutraceutical compounds, each selectively targeting distinct pathophysiological mechanisms implicated in the disease process, has been proposed [15]. Consequently, this randomized, single-blind, pilot study sought to explore and compare the efficacy and safety of two commercially available multi-ingredient supplements, featuring various combinations of HA, Glc, and CS, on patients with mild-to-moderate knee OA. 

## Materials and methods

Patients

This was a randomized, single-blind, pilot study involving 51 consecutive patients (35 women and 16 men) aged 40−75 years diagnosed with knee OA according to the American College of Rheumatology criteria [17]. Eligibility was determined based on specific inclusion criteria, which required participants to have experienced symptoms for a minimum of one month, a pain level of at least 2 on a visual analog scale (VAS) while at rest, morning stiffness lasting no longer than 30 min, and a synovial fluid leukocyte count of less than 2000/mL [18]. Pregnant or breastfeeding women were excluded, as were subjects who had a history of significant renal, hepatic, cardiac, gastrointestinal, or hematologic disorders, had neurologic or psychiatric conditions, were diagnosed with malignancies, suffered from atopy or allergic disorders, had type 2 diabetes or other endocrine disorders, experienced coagulation disturbances, had used NSAIDs within the two weeks leading up to the study, or had been treated with corticosteroids within the four weeks before the study. Based on the Kellgren-Lawrence Grading System [19], 58.8% (n = 30) and 41.2% (n = 21) of the study patients were classified with scores of 1 and 2, respectively, indicating mild-to-moderate knee OA. Informed consent was obtained from all participants, and the research protocol was approved by the local ethics committee (reference number 24/JHS).

Chondroprotective supplements

This investigation evaluated two commercially available, multi-ingredient chondroprotective supplements in tablet form: Syalox® 300 Plus (River Pharma, Orio Litta, Italy) and Cartijoint® Forte (Fidia Farmaceutici, Abano Terme, Italy). Syalox® 300 Plus comprises 300 mg of HA and 100 mg of Boswellia serrata extract. In contrast, Cartijoint® Forte is formulated with 415 mg of Glc, 400 mg of CS, and 50 mg of curcuminoids derived from the rhizomes of Curcuma longa L. These two supplements were selected for evaluation due to their widespread use and popularity as over-the-counter products for managing OA symptoms in Southern Europe.

Procedures

The duration of the study was set at four weeks. At baseline, body mass index (BMI) and the duration of experienced pain were recorded for all participants. Patients (n = 51) were allocated equally (1:1:1 ratio) into three groups: a control group (n = 17) that received no supplements (watchful waiting), an HA group (n = 17) given Syalox® 300 Plus (1 tablet/day), and a Glc + CS group (n = 17) given Cartijoint® Forte (1 tablet/day). The process of random allocation was facilitated by a computer-generated sequence. We used a single-blind design where all physicians and laboratory personnel were kept unaware of the supplement assigned to each patient, whereas the patients themselves were not blinded. All participants were instructed to refrain from engaging in other treatments for the entire duration of the study. Evaluations were conducted at two points: at baseline and upon completion of the four-week supplementation period.

Outcome measures

The primary outcome measure was the evaluation of changes from baseline to the study conclusion in patient-reported pain at rest and during movement, clinically measured range of motion, and overall patient-reported WOMAC scores [20]-including its three subscales assessing pain, stiffness, and physical function [20]-in the HA and Glc + CS groups. Additionally, we evaluated serum adropin levels, a peptide hormone recognized for its potential as an emerging biomarker in identifying knee OA due to its characteristically low levels in affected individuals [21].

Efficacy

The efficacy endpoints encompassed [18]: 1) patient-reported arthritis pain score at rest, measured via the VAS with a range of 0 to 10, where 0 signifies no pain and 10 represents the most severe pain; 2) patient-reported arthritis pain score during movement, assessed through the VAS with a range of 0 to 10, where 0 indicates no pain and 10 denotes the most severe pain; 3) range of motion, quantified in degrees; and 4) the Western Ontario and McMaster Universities Osteoarthritis Index (WOMAC) score [20]. The WOMAC index is comprised of subscales evaluating pain (0−20 scale, with 0 indicating no pain and 20 the most severe pain), stiffness (0−8 scale, with 0 representing no stiffness and 8 the most severe stiffness), and physical function (0−68 scale, with 0 indicating optimal functioning and 68 the poorest functioning). The aggregate WOMAC score spans from 0 to 96 [20].

Safety

Safety evaluations involved documenting any adverse events that emerged post-treatment, along with alterations from the initial baseline in clinical laboratory tests, vital signs, and physical assessments. Significant deviations in laboratory test results were defined according to the following criteria: increases in aspartate aminotransferase (AST) and/or alanine aminotransferase (ALT) to levels three times or more above the normal upper limit, creatinine levels exceeding 1.3 times the upper limit of normal, blood urea nitrogen (BUN) levels more than double the upper limit of normal, a reduction in hematocrit of 5 percentage points or more from the baseline, and a decrease in hemoglobin of 2 g/dL or more from the baseline measurement [18].

Measurements of serum adropin levels

Venous blood samples were collected into serum separator tubes at baseline and upon completion of the study. The blood was left to clot at ambient temperature for 30 minutes before being centrifuged at 1,000 × g for 15 min. The serum was then extracted, aliquoted, and stored at −80°C until analysis. Serum adropin concentrations were quantified using an enzyme-linked immunosorbent assay kit (Cusabio Biotech Co., Ltd., Wuhan, China) in accordance with the manufacturer’s instructions. The assay sensitivity threshold was 1.56 pg/mL, whereas the intra-assay and inter-assay coefficients of variation were 8% and 10%, respectively. To ensure unbiased results, all samples were analyzed simultaneously at the study’s conclusion by laboratory staff blinded to the clinical data.

Data analysis

The Kolmogorov-Smirnov test revealed that all continuous variables adhered to a normal distribution. Based on these findings, only parametric statistical methods were used for subsequent data analysis. Continuous variables are presented as means ± standard deviations and were analyzed using one-way analysis of variance (ANOVA), followed by post hoc Newman-Keuls tests for multiple comparisons. Categorical data are expressed as counts and percentages and were compared using the chi-square test. All analyses were performed using IBM Corp. Released 2011. IBM SPSS Statistics for Windows, Version 20.0. Armonk, NY: IBM Corp., with a two-sided significance level set at 5%.

## Results

General characteristics of the study participants

Table 1 summarizes the baseline characteristics of the three study groups. There were no significant intergroup differences regarding age, sex, BMI, duration of pain, pain intensity at rest and upon movement, range of motion (measured in degrees), and WOMAC scores. All laboratory safety parameters were within the normal range at baseline (data not shown). Therefore, the study sample can be considered representative of a clinical population of patients with mild-to-moderate OA.

**Table 1:** Baseline characteristics of the three study groups HA: hyaluronic acid; Glc: glucosamine; CS: chondroitin sulfate; VAS: visual analogue scale; WOMAC: Western Ontario and McMaster Universities.

Variable	Control group (n = 17)	HA group (n = 17)	Glc + CS group (n = 17)	p-value
Age, years	56.2 ± 4.4	56.5 ± 4.3	56.3 ± 4.1	0.72
Women/men	11/6	12/5	12/5	0.91
Body mass index, kg/m^2^	25.4 ± 2.2	25.9 ± 2.4	25.7.0 ± 23	0.52
Pain duration, months	3.0 ± 0.6	3.1 ± 0.8	3.2 ± 0.6	0.64
Pain at rest, VAS (0-10)	2.7 ± 0.4	2.8 ± 0.3	2.7 ± 0.4	0.76
Pain on movement, VAS (0-10)	3.6 ± 0.5	3.7 ± 0.6	3.7 ± 0.5	0.82
Range of motion (degrees)	136 ± 14	135 ± 13	136 ± 15	0.71
WOMAC scores				
Pain	5.7 ± 0.5	5.9 ± 0.7	5.8 ± 0.6	0.65
Stiffness	1.3 ± 0.2	1.4 ± 0.3	1.4 ± 0.4	0.71
Physical function	20.5 ± 2.6	20.8 ± 2.4	20.7 ± 2.5	0.78
Total	27.5 ± 3.5	28.1 ± 3.4	27.9 ± 3.6	0.62

Efficacy

All participants completed the study. Table 2 presents the efficacy outcomes for the three groups. As expected, the control group, which was subject to watchful waiting, did not exhibit any significant temporal changes. In contrast, both the HA and Glc + CS groups demonstrated significant improvements at the end of the study compared to baseline (all p < 0.05) about pain at rest and upon movement, range of motion, total WOMAC scores, and its three subscales (pain, stiffness, and physical function). However, the HA group demonstrated superior outcomes compared to the Glc + CS group in terms of improvement in pain at rest, pain upon movement, and the WOMAC pain subscale (all p < 0.05). No significant intergroup differences were observed concerning the range of motion (measured in degrees) and WOMAC stiffness and physical function subscales.

**Table 2:** Temporal course of clinical endpoints in the three study groups HA: hyaluronic acid; Glc: glucosamine; CS: chondroitin sulfate; VAS: visual analogue scale; WOMAC: Western Ontario and McMaster Universities. The data are expressed as means and standard deviations. *P  <0.05 versus baseline. †P < 0.05 versus the Glc + CS group

	Control group (n = 17)		HA group (n = 17)		Glc + CS group (n = 17)	
	Baseline	End of the study	Baseline	End of the study	Baseline	End of the study
Pain at rest, VAS (0-10)	2.7 ± 0.4	2.8 ± 0.2	2.8 ± 0.3	1.9 ± 0.5*,†	2.7 ± 0.4	2.3 ± 0.4 *
Pain on movement, VAS (0-10)	3.6 ± 0.5	3.4 ± 0.6	3.7 ± 0.6	2.7 ± 0.7*,†	3.7 ± 0.5	3.2 ± 0.4*
Range of motion (degrees)	136 ± 14	131 ± 13	135 ± 13	160 ± 17*	136 ± 15	157 ± 18*
WOMAC scores						
Pain	5.7 ± 0.5	5.8 ± 0.6	5.9 ± 0.7	4.4 ± 1.1*,†	5.8 ± 0.6	5.1 ± 1.0*
Stiffness	1.3 ± 0.2	1.4 ± 0.3	1.4 ± 0.3	1.1 ± 0.2*	1.4 ± 0.4	1.2 ± 0.3*
Physical function	20.5 ± 2.6	21.4 ± 2.3	20.8 ± 2.4	16.2 ± 2.2*	20.7 ± 2.5	16.4 ± 2.0*
Total	27.5 ± 3.5	28.6 ± 3.2	28.1 ± 3.4	21.7 ± 2.8*	27.9 ± 3.6	22.7 ± 2.5*

Safety

Treatment-emergent adverse events in both the HA and Glc + CS groups were infrequent and did not show any significant differences between the two groups (Table 3). No clinically relevant changes in laboratory values were observed in any of the study participants, regardless of the treatment group they were assigned to (data not shown).

**Table 3:** Treatment-emergent adverse events were observed in the two treatment arms HA: hyaluronic acid; Glc: glucosamine; CS: chondroitin sulfate. Data are expressed as the number of patients (percentages in parentheses).

	HA group (n = 17)	Glc + CS group (n = 17)
Gastrointestinal adverse events		
Dyspepsia	1 (5.9%)	1 (5.9%)
Nausea	0 (0%)	0 (0%)
Constipation	0 (0%)	0 (0%)
Diarrhea	0 (0%)	0 (0%)
Flatulence	2 (11.8%)	1 (5.9%)
Other adverse events		
Headache	0 (0%)	0 (0%)
Dizziness	0 (0%)	0 (0%)
Pruritus	0 (0%)	1 (5.9%)

Serum adropin levels

Table 4 presents a summary of the changes in serum adropin levels from baseline to the conclusion of the study for the three study groups. In the control arm, no significant differences were observed over time. Both the HA group and the Glc + CS group demonstrated a statistically significant increase in serum adropin levels compared to their respective baseline values (p < 0.05). However, the increase in serum adropin levels was significantly more pronounced in the HA group compared to the Glc + CS group (p < 0.05).

**Table 4:** Temporal course of serum adropin levels in the three study groups HA: hyaluronic acid; Glc: glucosamine; CS: chondroitin sulfate. *p < 0.05 versus baseline. †p < 0.05 versus the Glc + CS group.

	Control group (n = 17)		HA group (n = 17)		Glc + CS group (n = 17)	
	Baseline	End of the study	Baseline	End of the study	Baseline	End of the study
Serum adropin levels, pg/mL	52 ± 22	55 ± 18	48 ± 24	71 ± 34*,†	51 ± 29	63 ± 28*

## Discussion

In this comparative study of two multi-ingredient chondroprotective supplements, patients with mild-to-moderate knee OA experienced significant symptom relief when treated with either HA or a combination of Glc + CS, outperforming the control group. This was evidenced by significant improvements in pain at rest and during movement, increased range of motion, and better total WOMAC scores, including its subscales assessing pain, stiffness, and physical function. Notably, HA was superior to Glc + CS in alleviating pain at rest, pain during movement, and in the WOMAC pain subscale. The enhanced efficacy of HA was associated with a more substantial increase in serum adropin levels compared to that observed with Glc + CS. This finding suggests a potential association between the improved clinical outcomes with HA supplementation and its correlation with higher serum adropin, a biomarker linked to low-grade inflammation [21]. Nevertheless, additional studies are required to determine whether a causal relationship exists between HA supplementation, serum adropin levels, and clinical outcomes. While chondroprotective supplements are generally recognized as safe, it is important to acknowledge that they may pose certain risks, particularly hypersensitivity reactions in susceptible individuals [22]. Nevertheless, treatment-emergent adverse events were rare throughout the study, and no severe adverse reactions necessitated participant withdrawal from supplement use. Furthermore, clinical laboratory safety assessments demonstrated no notable alterations in either treatment arm, underscoring the favorable safety profile of both supplements. 

Despite limited consensus, non-pharmacological approaches for managing mild-to-moderate knee OA are gaining increased attention [23]. While several multi-ingredient chondroprotective nutraceuticals have been developed [8,9], there is limited direct evidence comparing their clinical safety and efficacy. In this study, a supplement containing 300 mg of HA and 100 mg of *Boswellia serrata* extract was found to be more effective than another nutraceutical formulated with 415 mg of Glc, 400 mg of CS, and 50 mg of curcuminoids derived from *Curcuma longa* L. rhizomes in reducing pain at rest and upon movement, as well as improving WOMAC pain subscale scores. The superior performance of the HA and *Boswellia serrata *extract supplements may be attributed to the synergistic effects of increased synovial HA concentration, which enhances joint lubrication and shock absorption [24], and the potent anti-inflammatory properties of *Boswellia serrata* [25]. In addition to contributing to the viscoelastic properties of synovial fluid, HA may also exhibit anti-inflammatory and chondroprotective actions by modulating intra- and extracellular inflammation cascades [26], potentially contributing to its clinical efficacy in reducing joint pain and preserving cartilage health. Furthermore, a previous animal study demonstrated that administration of standardized *Boswellia serrata* extracts in a rat model of OA resulted in the suppression of inflammatory enzymes such as cyclooxygenase-2 and 5-lipoxygenase, leading to a significant reduction in prostaglandin E2 and leukotriene B4 levels [27]. In addition, this extract may prevent cartilage degradation by downregulating matrix metalloproteinases, thus impeding the breakdown of glycosaminoglycans within the articular extracellular matrix [27]. While Glc [11], CS [12], and curcuminoids [28] also have anti-inflammatory and cartilage-protecting effects, the specific combination and dosages of HA and *Boswellia serrata *extract in the study may have resulted in a more effective reduction of pain and improvement in WOMAC pain subscale scores.

Adropin, a peptide hormone consisting of 76 amino acids, has recently been associated with various metabolic and inflammatory processes [29]. Notably, circulating levels of adropin have been inversely correlated with markers of inflammation, such as tumor necrosis factor-alpha and the neutrophil-to-lymphocyte ratio, in patients with knee OA [21]. Our investigation revealed that clinical improvements in symptoms were accompanied by a significant elevation in serum adropin levels in both study groups. Notably, the greater alleviation of pain observed in patients treated with HA and *Boswellia serrata* extract corresponded with a more substantial increase in circulating adropin levels compared to those receiving Glc, CS, and curcuminoids. These findings suggest that certain chondroprotective ingredients may be more effective than others in modulating adropin levels and, consequently, in managing inflammation and pain associated with knee OA. This observation opens new avenues for preclinical research to elucidate the mechanisms by which HA, Glc, CS, *Boswellia serrata*, and curcuminoids may differentially modulate adropin expression. Furthermore, these results may prompt the development of optimized nutraceuticals that leverage adropin’s anti-inflammatory properties to improve outcomes in patients with knee OA.

This research is subject to several constraints that warrant discussion. The limited number of participants may lead to an exaggerated perception of the efficacy of the treatment [30]. Additionally, the use of continuous variables and participant-reported outcomes introduces the possibility of bias. We also recognize the potential limitations inherent in the single-blind study design. Patients' awareness of their treatment assignment could affect their behavior, adherence, and reporting of subjective outcomes, potentially leading to an overestimation of the intervention's beneficial effects. To minimize this bias, we carefully explained to patients the importance of honest reporting and consistent behavior, regardless of their assigned treatment. In addition, the study incorporated both objective clinical measures (such as range of motion) and biochemical markers (specifically, adropin levels). It is important to view this investigation as preliminary; the duration of follow-up was brief, and corroborative studies are necessary to substantiate and expand upon our findings. While the results demonstrate statistical significance, further exploration is needed to fully elucidate the clinical relevance and implications of these findings. Translating these outcomes into tangible benefits for patient care and treatment strategies warrants additional research and discussion to bridge the gap between statistical significance and practical clinical application. Management of OA of the knee should be tailored to the unique clinical profile of each patient, drawing upon evidence from clinical trials. Notably, we did not directly compare the efficacy of the dietary supplements to established pharmacological treatments for osteoarthritis, such as NSAIDs. Future research should examine how these supplements perform relative to standard medical therapies to provide additional clinical context for physicians and patients considering supplement use for OA management. To thoroughly evaluate the therapeutic potential of chondroprotective supplements for individuals with inflammatory joint conditions, additional research is needed. Future studies should include larger sample sizes, incorporate a placebo arm, or utilize a cross-over design to enhance the reliability and generalizability of the findings. It is also important to note that this study did not include a placebo control group, which limits our ability to definitively attribute the observed symptom relief to the supplements alone. The possibility of placebo effects contributing to the reported improvements cannot be ruled out without further placebo-controlled trials. Furthermore, while our study observed a favorable safety profile, confirmation in larger-scale investigations is necessary to bolster confidence in making broader safety claims. 

## Conclusions

This study demonstrates that oral supplementation with either HA or a combination of Glc + CS is a safe and effective approach for managing mild-to-moderate knee OA. Among the two, HA supplementation offers superior outcomes in terms of pain relief and elevation of serum adropin levels, a novel biomarker associated with cartilage metabolism and inflammation. Notably, the observed increase in serum adropin levels with HA supplementation opens new avenues for research into the mechanisms underlying OA and its treatment. Ultimately, this study contributes to the growing body of evidence supporting the use of nutritional supplements in the management of OA.
